# Cytokine levels in abdominal exudate predict prolonged postoperative ileus following surgery for colorectal carcinoma

**DOI:** 10.3892/ol.2013.1465

**Published:** 2013-07-15

**Authors:** PENGCHENG ZHU, HONGHUA JIANG, JIHONG FU, WEI CHEN, ZHONGCHUAN WANG, LONG CUI

**Affiliations:** Department of Colorectal Surgery, Xinhua Hospital Affiliated to Shanghai Jiaotong University School of Medicine, Shanghai 200092, P.R. China

**Keywords:** prolonged postoperative ileus, colorectal surgery, peritoneal cytokines, diagnostic predictor

## Abstract

The aim of the present study was to investigate whether the measurement of cytokines from abdominal exudate is valuable for the early diagnosis of prolonged postoperative ileus (PPOI) following colorectal surgery. In the present study, 100 consecutive patients who were scheduled to undergo elective resection for carcinoma of the sigmoid or rectum were investigated. Abdominal exudate was obtained via a drain tube following surgery for the detection of interleukin (IL)-1β, IL-6 and tumour necrosis factor (TNF)-α. The correlation among the cytokine levels on postoperative days 1, 3 and 5 and the development of PPOI was investigated. Eight patients developed PPOI which was diagnosed 10–15 days postoperatively. No significant differences were observed among the peritoneal cytokine levels on postoperative days 1 and 3 in the 8 patients who developed PPOI when compared with those of the 92 patients that did not develop PPOI. By contrast, cytokine levels on postoperative day 5 were significantly higher in patients who developed PPOI when compared with those of patients that did not develop PPOI. The cytokine levels significantly increased during the first 5 days postoperatively in patients who developed PPOI and significantly decreased in patients who did not develop PPOI. The results of the present study showed that the increase in peritoneal IL-1β, IL-6 and TNF-α levels may be an additional early diagnostic predictor of PPOI following colorectal surgery.

## Introduction

Postoperative ileus (POI) is described as a transient impairment of bowel motility that may occur following major surgery (1). The process of postoperative bowel recovery following abdominal surgery, including colectomy, typically lasts for 3–5 days (2). Prolonged postoperative ileus (PPOI) usually manifests with nausea, vomiting, abdominal pain and distension and/or delay in the passage of flatus and stool. PPOI is associated with prolonged hospitalization and readmission and high morbidity and mortality (3). Although PPOI is a severe condition that requires immediate diagnosis and appropriate treatment (4), the accurate and early diagnosis of PPOI remains difficult due to a lack of appropriate laboratory parameters (5). Early detection of PPOI is critical for early intervention and minimizing mortality.

Cytokines are pleiotropic substances with a diversity of functions (6) and the cytokines IL-1β, IL-6 and TNF-α are significant mediators of the acute-phase response in humans (6,7). It has been previously reported that peritoneal cytokines are sensitive indicators of the postoperative inflammatory reaction and may be predictors of serious surgical complications (8). In addition, monitoring peritoneal cytokine levels following elective gastrointestinal surgery may indicate severe intra-abdominal complications (9) and the present study investigated whether the measurement of peritoneal cytokines was an early predictor of PPOI following colorectal surgery.

## Patients and methods

### Patients

In the present study, 100 consecutive patients (60 male and 40 female) with carcinoma of the sigmoid or rectum and scheduled to undergo elective open anterior resection under general anesthesia between January and December 2011 were included. Of note, ileostomy was not perfomed on patients during surgery. Postoperative care for all patients included an analgesia protocol using a patient-controlled analgesia (PCA) pump of morphine chlorhydrate for 48 h and the dose of morphine chlorhydrate used was recorded. Exclusion criteria included patients who had received preoperative radiotherapy or chemotherapy, undergone emergency surgery and received anti-inflammatory drugs including corticosteroids and immunosuppressants preoperatively for infectious or inflammatory diseases. The mean age of the 100 eligible patients was 59 years (range, 24–87 years) at the time of surgery. The site of the carcinoma was the sigmoid colon in 26 patients and the rectum in 74 patients. All the patients underwent bowel resection with end-to-end anastomosis with the aid of circular staplers. Prior to the closure of the peritoneum, a silastic drainage tube was placed in the pelvic base of all the patients and removed 5–12 days following surgery. This study was approved by the ethics committee of Xinhua Hospital Affiliated to Shanghai Jiaotong University School of Medicine. Written informed consent was obtained from the patients.

### Definition of PPOI

POI is commonly observed ~3–5 days following major abdominal surgery and presents a number of clinical symptoms, including abdominal pain or distension, nausea or vomiting, the inhibited passage of feces and/or flatus and abnormal bowel sounds. POI develops in the majority of patients undergoing abdominal surgery and it is considered to be a normal phenomenon during the postoperative course. However, a previous study (1) reported that among patients who underwent abdominal surgery, 7% developed PPOI with the observation of clinical manifestations and radiological imaging for ileus, which usually required clinical intervention. In addition, findings of another study demonstrated that the recovery of small bowel motility and absorption occurs within hours of surgery, whereas gastric and colonic functions may require 2–5 days (10). An improved clinical definition of PPOI is an ileus that persists for >6 days following surgery with evident clinical symptoms and radiological imaging which must be diagnosed at an early stage for immediate treatment (11). This definition was utilized in the present study and therefore, patients were divided into two groups: PPOI (with evident clinical symptoms and radiological imaging of PPOI) and No-PPOI (with no clinical symptoms and radiological imaging of PPOI).

### Sampling

Samples of abdominal exudate were collected via abdominal drains on postoperative days 1, 3 and 5 for detection. Samples were obtained as fresh material within 2 h of the morning emptying of the drainage bag. Measurements of 5 ml abdominal exudate were collected into a vacutainer tube and immediately centrifuged at 1,400 × g for 15 min. Samples were stored at −80°C until analysis. The levels of IL-1β, IL-6 and TNF-α were measured by enzyme-linked immunosorbent assays (R&D Systems, Minneapolis, MN, USA). Each assay was performed in duplicate and a standard curve was constructed using recombinant cytokines. The minimum sensitivity of each cytokine assay was 2.0 pg/ml.

### Statistical analysis

Comparison of frequencies between the groups was analysed using the χ^2^ test with Yates' correction. Continuous data were presented as the mean ± SD. Mean values between the two groups were compared using the unpaired t-test. For comparisons involving more than two groups, a one-way analysis of variance was applied and changes with time were analysed using the paired t-test. P<0.05 was considered to indicate a statistically significant difference.

## Results

### Clinical characteristics

PPOI occurrence was clinically proven in 8/100 patients (8%; 6 male and 2 female) who underwent elective open resection for carcinoma of the sigmoid or rectum. The median number of days to PPOI diagnosis was 12 days (range, 10–15 days) following surgery. Conservative treatment was administered to 6/8 PPOI patients to promote bowel movement and the remaining 2 patients were urgently reoperated on for adhesiolysis. All 8 PPOI patients recovered well following treatment and there were no mortalities for the duration of the present study. No significant differences were observed among the majority of clinical variables, including age, gender, BMI, location of carcinoma, intraoperative blood loss, operative time, dose of morphine during PCA and duration of postoperative hospitalization in the No-PPOI group when compared with that of the PPOI group. However, the number of days to the first passage of flatus was significantly higher in the PPOI group when compared with that of the No-PPOI group (Table I).

### Peritoneal cytokines

**Figure 1:**
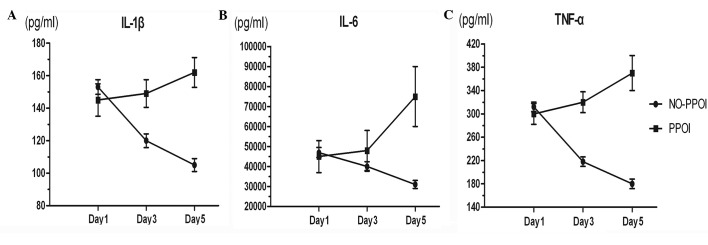
No significant differences were observed between the peritoneal cytokine levels on postoperative days 1 and 3 and patients who developed PPOI when compared with those that did not develop PPOI (day 1: IL-1β, P=0.23; IL-6, P=0.35 and TNF-α, P=0.62 and day 3: IL-1β, P=0.34; IL-6, P=0.65 and TNF-α, P=0.29). By contrast, cytokine levels on postoperative day 5 were significantly higher in patients who developed PPOI when compared with patients that did not develop PPOI (IL-1β, P=0.012; IL-6, P<0.0001 and TNF-α, P<0.0001). Cytokine levels significantly increased in the first 5 days following surgery in patients who developed PPOI (IL-1β, P=0.045; IL-6, P=0.03; TNF-α, P=0.02) and significantly decreased in patients who did not develop PPOI (IL-1β, P<0.0001; IL-6, P<0.0001; TNF-α, P<0.0001). IL, interleukin; TNF, tumour necrosis factor; PPOI, prolonged postoperative ileus. Data are presented as the mean ± SD.

The values of IL-1β, IL-6 and TNF-α levels on postoperative days 1, 3 and 5 are shown in Table II. The impact between patient demographic factors on day 1 and peritoneal cytokine production was also examined (Table III). Age (<60/≥60 years), gender, normal or poor (>10% loss of body weight) preoperative nutritional status, site of carcinoma (sigmoid colon/rectum) and the Dukes' stage (A/B/C) did not affect the peritoneal cytokine levels. The changes in peritoneal cytokine level for a 5-day duration following surgery are shown in Fig. 1. No significant differences were noted on postoperative days 1 and 3 among the levels of IL-1β, IL-6 and TNF-α and the 8 patients who developed PPOI when compared with those of the 92 patients that did not develop PPOI. However, on postoperative day 5 the levels of IL-1β, IL-6 and TNF-α were significantly higher in patients who developed PPOI when compared with those of patients who did not develop PPOI. The levels of IL-1β, IL-6 and TNF-α decreased in the 92 patients without PPOI and significantly increased in the 8 patients with PPOI. During the first 5 days following surgery the IL-1β levels increased in 7/8 patients (88%) with PPOI vs. 16/92 patients (17%) without PPOI (P=0.0001), the IL-6 levels increased in 8/8 patients (100%) with PPOI vs. 15/92 patients (16%) without PPOI (P<0.0001) and the TNF-α levels increased in 8/8 patients (100%) with PPOI vs. 13/92 patients (14%) without PPOI (P<0.0001).

## Discussion

Acute bowel obstruction is a major cause of morbidity, increased hospitalization costs and emergency conversion surgery (11,12). Diagnosis of bowel obstruction traditionally occurs via classic signs including tachycardia, hypotension, fever, constant pain, peritoneal signs, leukocytosis, base deficit and metabolic acidosis (13) and abdominal CT (14). The immediate and appropriate treatment of this condition is critical, however, there are no accurate diagnostic methods or tools currently available (15). Although the combined evaluation of clinical, laboratory and radiological observations is recommended, the preoperative early diagnosis of bowel obstructions cannot be made or excluded reliably by any identified parameters, individual or combined or even by experienced clinical judgement (16).

The aim of the present study was to evaluate the predictive value of peritoneal cytokines for the early diagnosis of bowel obstructions in patients following surgery for colorectal carcinoma. Although the value of plasma cytokines for the diagnosis of PPOI has been evaluated in previous studies (17), it has not been investigated in abdominal exudate.

Peritoneal cytokines respond extensively following abdominal surgery and therefore must be a significant marker of postoperative complications (9). The IL-1β, IL-6 and TNF-α levels were measured on postoperative days 1, 3 and 5 to investigate the correlation between changes in cytokine levels and the development of PPOI. In the normal postoperative progression, the peak of peritoneal cytokine production is observed on postoperative day 1 and subsequently decreases with time (18). The increased levels of peritoneal cytokines, prior to marked clinical symptoms, must be a significant marker to predict future intraabdominal ileus.

Soybel and Zinner (19) reported that ileus is correlated with the increased expression of local inflammatory cytokines and chemokines, leukocyte infiltration into the muscularis and the release of mediators from resident and infiltrating leukocytes. These mediators are capable of directly inhibiting the contraction of intestinal smooth muscle and subsequent progression of ileus.

A systemic cytokine network has been suggested to induce impaired gastric electrical activity with ileus status (20). The local inflammatory status may have a significant modulating role and correlations between ileus and increased systemic levels of proinflammatory cytokines (IL-1β, IL-6 and TNF-α) were also identified. Previous studies (19) have revealed that the dense network of macrophages specific to the muscularis is rapidly activated during impaired intestinal motility, thus initiating an inflammatory cascade of events, including the upregulation of cytokines, chemokines and kinetically active substances (21). This local inflammatory status results in the recruitment and extravasation of leukocytes into the circular muscle layer. In conjunction with an increased expression of adhesion molecules, leukocytes inflitrate through the muscularis externa. The secretion of various potent leukocytic products directly succeeds and inhibits the contraction of gastrointestinal smooth muscle.

At present, a marked number of clinical studies have demonstrated the significance of cytokines as an early and reliable diagnostic and prognostic tool in several conditions, including infections, infectious and non-infectious SIRS, sepsis, acute pancreatitis and other postoperative complications (22). However, compared with that of systemic (plasma) cytokines, peritoneal cytokines respond extensively following major abdominal surgery, indicating that the measurement of peritoneal cytokines is a likely method to determine postoperative inflammatory reaction (8) which may progress to POI. In future studies, the preoperative systemic, as well as postoperative systemic and peritoneal cytokine measurements are likely to be performed together to investigate whether the cytokines in the peritoneal fluid are affected by the systemic circulation. In addition, the systemic and peritoneal cytokine levels following surgery are likely to be compared to indicate which is the most suitable for determining PPOI.

Results of the present study demonstrated that the estimation of cytokine levels from abdominal exudate may prove to be an early diagnostic tool that is likely to support the decision-making process of surgeons for the early detection of PPOI following colorectal surgery. The measurement of peritoneal cytokines during the early postoperative period functions as a useful diagnostic marker to predict future PPOI. Therefore, it must allow for the appropriate therapeutic intervention to minimize PPOI-related morbidity, similar to that of the use of biologics to block cytokine action.

## Figures and Tables

**Table I:** Data of patients receiving anterior resection of the sigmoid or rectum.

Variables	No-PPOI	PPOI	P-value
Age, years	61.6±17.6	53.37±8.41	0.192
Gender			0.471
Male	54	6	
Female	38	2	
BMI	23.62±4.62	25.87±4.48	0.213
Site of carcinoma			0.160
Sigmoid colon	24	2	
Rectum	68	6	
Blood loss, ml	186.32±12.17	272.86±78.40	0.315
Operative time, min	164.68±30.62	184.43±32.60	0.447
Dose of morphine during PCA, mg	188.22±117.61	194.84±127.30	0.852
Time to first passage of flatus, days	3.09±1.09	10.00±1.41	0.001
Postoperative hospital stay, days	15.62±4.46	35.43±12.21	0.043

PCA, patient-controlled analgesia; PPOI, prolonged postoperative ileus. Data are presented as the mean ± SD.

**Table II:** Peritoneal cytokine levels on postoperative days 1, 3 and 5.

Day	IL-1β (pg/ml)	IL-6 (pg/ml)	TNF-α (pg/ml)
1	152±5.4	52,000±2600	312±10.7
3	126±4.8	40,000±2500	218±8.9
5	86±5.1	36,700±2100	197±9.2

IL, interleukin; TNF, tumour necrosis factor. Data are presented as the mean ± SD.

**Table III:** Peritoneal cytokine production level on postoperative day 1 vs. patient demographic factors.

Variables	n	IL-1β (pg/ml)	IL-6 (pg/ml)	TNF-α (pg/ml)
Age, years				
<60	54	149±6.4	44000±2,800	306±12
≥60	46	157±5.6	53,200±2,900	316±11
P-value		0.78	0.66	0.51
Gender				
Male	60	157±7.7	54,200±2,600	298±12
Female	40	149±6.8	52,000±2,800	325±10
P-value		0.82	0.53	0.23
Preoperative nutritional status				
Normal	82	150±7.2	52,400±2,500	312±11
Poor	18	156±7.5	50,800±5,100	316±17
P-value		0.47	0.89	0.72
Site of carcinoma				
Sigmoid colon	26	148±5.2	49,800±2,800	314±13
Rectum	74	151±6.2	52,100±3,700	307±12
P-value		0.62	0.34	0.45
Dukes' stage				
A	16	148±8.9	48,600±5,300	314±19
B	37	151±7.4	54,300±4,100	309±13
C	47	144±6.2	51,000±4,400	311±15
P-value		0.91	0.94	0.88

IL, interleukin; TNF, tumour necrosis factor. Data are presented as mean ± SD.
